# Glutathione S-transferase *CrGST24* in the differentiation of adventitious buds from *Camellia reticulata* callus

**DOI:** 10.3389/fpls.2025.1641401

**Published:** 2025-08-15

**Authors:** Zihang Cao, Jianzhao Wang, Yikai Gong, Xian Yin, Cheng Yang, Ting Yang, Tian Wu

**Affiliations:** Engineering Technology Research Center of National Forestry and Grassland Administration on Southwest Landscape Architecture, Yunnan Province Engineering Research Center for Functional Flower Resources and Industrialization, College of Landscape and Horticulture, Southwest Forestry University, Kunming, China

**Keywords:** *Camellia reticulata*, *CrGST24*, auxin, cytokinin, GSH, ASA, ROS

## Abstract

**Introduction:**

*Camellia reticulata* holds cultural and horticultural significance in traditional Chinese gardens, as a regional endemic species in Yunnan Province. However, during the *in vitro* regeneration process of *C. reticulata*, there is often a phenomenon of low efficiency of adventitious bud differentiation or no adventitious bud differentiation at all.

**Methods:**

In previous study, we observed significant morphological differences between the callus tissues of *C. reticulata* ‘Zipao’ and wild species. The callus of ‘Zipao’ was white and loosely textured, the wild species was green and hard. To investigate the differences between these two types of callus, we conducted transcriptome analysis and identified a differentially expressed gene *GST24*, which is closely related to the synthesis of glutathione (GSH). Heterologous transformation of *CrGST24* into tobacco (*Nicotiana tabacum*) was conducted.

**Results and discussion:**

The *CrGST24* gene promoted the synthesis of endogenous auxin and cytokinin in transgenic tobacco by regulating the expression of transcription factors related to auxin and cytokinin. Exogenous IBA, 6-BA, and red-blue light treatment increased the levels of auxins and cytokinins in *C. reticulata* callus, thereby promoting adventitious bud differentiation. Additionally, *CrGST24* interacted with *CrGSHB* and *CrDHAR2* genes, facilitating GSH and AsA synthesis and clearing ROS.

## Introduction

*Camellia reticulata*, also known as Yunnan camellia is among the decorative trees belonging to the Camellia genus, which are members of the Theaceae family. China is considered the origin and distribution hub of Camellia. Generally, members of the genus Camellia are classified into one of five groups. Yunnan camellia (*C. reticulata*) and its near relatives constitute a significant group that is primarily found in Yunnan Province. In Yunnan province, *C. reticulata* was regarded as an exceptional ornamental plant with high economic values. Some of them are very important flowering ornamentals that also produce oil, and have a wide variety of cultivars. In addition to its cultural and medicinal significance, *C. reticulata* is also a valuable source of food products. *C. reticulata* is mostly red-colored, and there are few accounts of color variations in the species. As a result, producers and researchers have constantly sought to breed *C. reticulata* of diverse colors to enhance its aesthetic value (Dai et al., 2013; Tong et al., 2015). *C. reticulata* is of great significance in ornamental, medicinal, and economic value. However, in breeding, its callus often has trouble differentiating into adventitious buds or may not differentiate at all.

Glutathione-S-transferase (GST) is extensively involved in various physiological and metabolic processes in plants and plays a key regulatory role when plants are exposed to stresses such as oxidation, pathogens and heavy metals. *GST* gene plays an important function in plant growth and development, and *GST* can be induced by phytohormones such as auxin, cytokinin, SA (Salicylic acid), MJ (Methyl jasmonate), and ETH (Ethylene). In tomato, the *SlGST43* gene plays an extremely important role in scavenging ROS. It interacts with *SlCOMT2* to regulate lignin content. Further studies indicate that *SlMYB71* and *SlWRKY8* interact to enhance the expression of *SlGST43* (Yuan et al., 2024). In tomato, overexpression of the *SlGST38* gene increased tomato resistance to viruses, and when *SlGST38* was knocked out, tomato rapidly became infected with viruses, and a large accumulation of ROS resulted in tomato mortality (Méndez et al., 2023). *GST* genes were auxin binding proteins and influenced their function by regulating the redox state of auxin, and *GST* genes were involved in auxin signalling through non-catalytic effects. In addition, *GST* genes may regulate the concentration and gradient of auxin in plants by binding to auxin (Abel and Theologis, 1996). *GST24*, *GST25*, and *GST26* genes were significantly co-expressed with the cytokinin signalling pathway, and *GST24*, *GST25*, and *GST26* had important roles in cytokinin signalling (Pavlů et al., 2018). GSH acted as an antioxidant by bursted ROS and participated in the ascorbate-glutathione cycle that eliminates peroxides (Rouhier et al., 2008). GSH is a combination of glutamic acid, cysteine, and glycine. GSH is often divided into oxidised glutathione (GSSG), and reduced glutathione (GSH). Auxin induced the expression of glutathione synthetase (GS), and glutathione S-transferase (GST), thereby increased glutathione synthesis (Kong et al., 2015). Exogenous application of auxin significantly increased glutathione levels and enhanced antioxidant capacity in *Arabidopsis thaliana* (Zechmann et al., 2011). Ascorbic Acid (AsA), also known as Vitamin C, it had an extremely important role in antioxidant and boosting plants against biotic and abiotic stresses. In tomato seedling root systems, exogenous growth hormones and cytokinins were applied to increase AsA levels and enhance plant resistance to environmental stress (Ahanger et al., 2018). AsA affected gene expression in the auxin synthesis pathway by interacted with the auxin response factor *ARF4*, *ARF4* promoted AsA accumulation by transcriptionally repressed *MYB99*, which in turn activated the expression of the AsA synthesis genes *GPPS*, *GLDH* and *DHAR* (Xu et al., 2023). However, research on the *GST* gene in *C. reticulata* is limited. The relationship between the *GST* gene and plant hormones, as well as the pathways through which the *GST* gene promotes the synthesis of GSH and AsA, requires further investigation.

In this study, we screened a *GST* gene from *C. reticulata* callus transcriptome data (Accession PRJNA909456 and Submission ID SUB12368224 at the NCBI BioProject database). *C. reticulata* callus were inoculated in medium containing different concentrations of IBA and 6-BA and placed under different light environments to analyse the expression of *GST* genes. The *GST* gene was heterologously transformed into tobacco and the function of transgenic tobacco was analysed. Finally, this experiment aimed to investigate experimental conditions that would be most conducive to the differentiation of adventitious buds from *C. reticulata* callus.

## Materials and methods

### Plant materials and growth conditions

The plant materials were *C. reticulata* ‘Zipao’ and wild species callus. ‘Zipao’ callus were white and loose (**Supplementary Figure 1a**); callus of the wild species were green and hard (**Supplementary Figure 1b**). Tobacco was used as the model plant for heterologous genetic transformation. Tobacco and *C. reticulata* callus were cultivated in a tissue culture room maintained at a constant 25°C, with a daily light exposure of 10-12 h and a humidity range of 65-75%, light intensity was 5000 LX. The red and blue light intensity required for subsequent experiments were 5000 LX.

### The *CrGST24* gene cloning

RNA was isolated using Vazyme Biotech RNA extraction kit (Nanjing, China), according to manufacturer’s protocol. Utilizing the NCBI online platform, specific cloning primers P1-F (AAGCAGTGGGAGTAGAGGTG) and P1-R (TTTGGCAAAGGCGAGTAGTT) were designed. The cDNA synthesized via reverse transcription was employed as the template to amplify the full-length sequence of *CrGST24*. The RT-PCR parameters were established as: 1 cycle of 5 min at 94°C, 35 cycles of 30 s at 94°C, 35 cycles of 30 s at 58°C, 35 cycles of 1 min at 72°C, followed by 1 cycle of 10 min at 72°C and 1 cycle of 10 min at 10°C, within a 50 μL reaction buffer. Subsequently, a gel recovery kit (Biomed, Beijing, China) was applied to purify the target bands. Eventually, the purified target band was ligated into a cloning vector and introduced into *E. coli* recipient cells, and then the plasmid isolated and sequenced.

### Expression analysis of *CrGST24* under different concentrations of IBA and 6-BA, different light environments and different light quality conditions

Three culture medium YS2, YS3, and YS4 containing different concentrations of IBA and 6-BA were prepared by increasing the concentrations of IBA and 6-BA individually or simultaneously on the basis of YS1 medium (**Supplementary Table 1**). Callus of *C. reticulata* ‘Zipao’ and wild species were inoculated on four different culture media and cultured under white light for 2 h and one week. The 2^-(ΔΔCt)^ method was used to analyse the expression of *CrGST24* gene in different types of healing tissues and under different concentrations of IBA and 6-BA treatments.

The callus of *C. reticulata* ‘Zipao’ and wild species were inoculated onto YS1 medium and put into light and dark environment for 8h. The 2^-(ΔΔCt)^ method was used to analyse the expression of *CrGST24* gene in different light environments.

The callus of *C. reticulata* ‘Zipao’ and wild species were inoculated on the YS1 medium and placed under white, red, and blue light for 8 h and three weeks. The 2^-(ΔΔCt)^ method was used to analyse the expression of *CrGST24* gene in conditions with white, red, or blue light.

### Overexpression vector construction, plant transformation of *CrGST24*, and identification of transgenic lines

The *CrGST24* gene was subjected to double digestion using the restriction enzymes *BamHI* and *PstI*. Agarose gel electrophoresis, followed by gel cutting and DNA recovery with the TAKARA Gel DNA Recovery Kit (Beijing, China), linearized the cloning vectors. The cleaved product was purified *via* a DNA purification kit. The target fragment was inserted into the pCAMBIA1300-35S vector. This construct was introduced into *E. coli* to screen positive clones and conduct sequencing. Finally, the extracted plasmids were transferred into *Agrobacterium tumefaciens* GV3101.

The heterologous genetic transformation of tobacco *via Agrobacterium* was performed according to Wang et al. (2024). Ten transgenic lines were randomly selected. RNA was extracted from the transgenic tobacco following the RNA extraction kit’s instructions, reverse transcribed into cDNA, and then verified by qPCR to screen for high-expressing positive lines.

Phenotypic analysis was conducted on high expressing transgenic tobacco lines and WT tobacco to observe any phenotypic differences between them.

### *In vitro* regeneration growth of *CrGST24* transgenic tobacco

WT tobacco and *CrGST24* transgenic tobacco leaves were cut into 0.5 cm*0.5 cm leaf discs and inoculated into normal MS medium, which was observed at 5 d intervals to analyse the *in vitro* regeneration of transgenic tobacco leaves.

### Expression analysis of auxin and cytokinin related genes in transgenic tobacco

The NCBI Primer-Blast was used to design qPCR primers for the growth hormone-related genes *NtYUCCA8*, *NtPIN1c*, *NtGH3.3*, *NtIAA27*, *NtARF6*, *NtWOX5*, and *NtSAUR20*; and for the cytokinin-related genes *NtARR5*, *NtARR12*, *NtCRE1*, *NtAHP6*, *NtCRF6*, and *NtWUS* qPCR primers, the *NtEF1α* as a reference gene (**Table 1**).

**Table 1:** qPCR primers.

Gene Name	Forward Primer (5’-3’)	Reverse Primer (5’-3’)	Description
*NtEF1α*	TGGTTGTGACTTTTGGTCCCA	ACAAACCCACGCTTGAGATCC	Reference gene
*NtYUCCA8*	ATGTGTATGGGTAAATGGTCC	CAGATTTTTCCAAGATTACAC	Involvement in auxin synthesis
*NtPIN1c*	GCAATGGCTGTGAGATTC	TGTAGTAGACCAGTGTTATCG	Involvement in the polar transport of auxin
*NtGH3.3*	GAAGGAATCATCACGAGAAT	GTCAACAAGGTCAACAAGA	Involvement in auxin synthesis
*NtIAA27*	AGAATGTACTAACCGTCCAA	CTCCATCCTTGTCTTCGTA	Involvement cell division and elongation
*NtARF6*	GCTCCTTACTATCTCCTACC	TCACAATCGCTACCAACT	A key role in auxin signal transduction
*NtWOX5*	CTCCTGCCAAGACAATAATATC	GCTTCTCTGACTCCGATT	A crucial role in processes of plant growth and development
*NtSAUR*	GTAGTGATTCAGACAGTTGTAG	TTGCCTAACGACCTCATT	An important role in plant growth and development
*NtARR5*	AAGTGACAGCAGTTGAGA	TCCAGGCATAGAATAATCAGT	A critical role in the cytokinin signaling pathway
*NtARR12*	AATGGACCTGCCTGTTAT	TGTTCTTCAATGCCTCAATG	A critical role in the cytokinin signaling pathway
*NtCRE1*	GATACCTGCTTGGATGGA	GACGAGACACTTGCTAATG	Involvement in auxin and cytokinin signal transduction
*NtAHP6*	GCCTGTAAGATTACGAATGT	TCCAACTTGCTCTGAAGA	Involvement in the synthesis of cytokinin
*NtCRF6*	AGAAGAAGAATCGTCGGCGG	GGTCAGGGGCAATTTCGGTA	A crucial component of the cytokinin signaling pathway
*NtWUS*	CCACCACAAGTATAACAACA	GTAGAATCCGCCTGAAGA	A crucial role in processes of plant growth and development

### Expression analysis of auxin and cytokinin related genes in *C.reticulata*

Twelve auxin and cytokinin related genes were screened from the callus transcriptome data of *C. reticulata* and their expression was analyzed

### Determination of auxin, cytokinin, ROS, GSH, and AsA contents in *CrGST24* transgenic tobacco

Auxin and cytokinin content in transgenic tobacco and in WT were examined by ELISA enzyme-linked immunoassay. Absorbance values of auxin and cytokinin should be measured at 450 nm.

Preparation of NBT staining solution: Dissolve 50 mg NBT in 100 mL Tris buffer (pH: 7.4), and obtain NBT staining working solution after full dissolution. The prepared staining solution needs to be stored at 4°C away from light. The leaves of high expression positive transgenic tobacco and WT were cut off, and the leaves were cut into circular leaf discs with a diameter of 2 cm using a hole punch, the cut circular leaf discs were immersed in NBT staining solution, and the leaf discs were taken out after being kept away from light for 48 h. The leaf discs were immersed in 95% alcohol, and the discs were decolored with stirring while heating, and the blue areas on the leaf discs after decolorization were the areas where the ROS were accumulated. ROS, GSH, and AsA content in transgenic tobacco and in WT were examined by ELISA enzyme-linked immunoassay. Absorbance values of ROS should be measured at 450 nm; absorbance values of GSH should be measured at 412 nm; absorbance values of AsA should be measured at 265 nm.

### A study on the interaction among *CrGST24*, *CrGSHB*, and *CrDHAR2*

Primary antibodies specific to the target protein were added and incubated, followed by incubation with secondary antibodies conjugated with fluorescent dyes to label the target protein, the nuclei were stained with DAPI. The coding sequences of *CrGST24*, *CrGSHB*, and *CrDHAR2* were fused with the green fluorescent protein (GFP) to construct the corresponding fusion protein expression vectors. These vectors were then transformed into tobacco leaf cells to achieve transient expression, enabling the synthesis of the fusion proteins within the plant cells. Finally, confocal laser scanning microscopy was used to observe the distribution of fluorescent signals in the tobacco leaf cells, so as to determine the subcellular localization of the target proteins.

The full-length CDS of the *CrDHAR2* (Accseeion No. PV920956 at the NCBI BioProject database) and *CrGSHB* (PV920957) were amplified and recombined into the pGADT7 vector to construct the prey vector. The *CrGST24* (PV920955) was intercepted and inserted into the pGBKT7 vector to construct the bait vectors. The prey and bait vectors were co-transferred into the Y2H Gold yeast strain as described in the instructions (Clontech, Shanghai, China). Yeast strains carrying the indicated vectors were plated on SD/-Leu-Trp growth medium. The positive clones were diluted with 0.9% (w/v) NaCl to OD_600_ of 0.2, after which 10 μL of each suspension was dropped on SD/-Leu-Trp, SD/-Leu-Trp-His-Ade with X-α-Gal medium. The experiments were performed three times with similar results.

The target gene’s DNA sequence was cloned into a vector containing the luciferase (LUC) reporter gene to form a recombinant plasmid. This plasmid was then transformed into tobacco leaf cells *via Agrobacterium*-mediated transformation and transiently expressed there. The luciferase detection system, with the substrate added to induce luminescence, was used, and the imaging system recorded the luminescent signals.

### Role of different concentrations of IBA, 6-BA, and different light qualities in the growth of *C. reticulata* callus

The *C.reticulata* ‘Zipao’ and wild species callus were placed in YS1, YS2, YS3 and YS4 callus medium, and they were placed in white light, dark, red and blue light environments cultured for 30 d.

The callus of *C.reticulata* ‘Zipao’ and wild species were inoculated onto YS3 medium and placed under red, blue, red-blue ratio of 2:1 and blue-red light ratio of 2:1 for one month.

### Role of GSH and AsA in the induction of adventitious shoots in *C. reticulata* callus

The *C.reticulata* ‘Zipao’ and wild species callus were put into AsA1, GSH1 and A1G1 liquid medium, and cultured at 28°C and 220 R for 48 h, then inoculated into YS3 medium, and cultured for 48 h with dark place, and then put into the light environment of blue-red light ratio of 2:1. Callus inoculated into YS3 medium and cultured in blue-red light ratio of 2:1 environment without any treatment were used as control.

### Method of making paraffin sections

The paraffin section method was used to observe the internal cellular structure of the callus of ‘Zipao’ and wild species *C. reticulata* after treatment with different light intensities, light qualities, GSH, and AsA. Making paraffin sections mainly includes: sampling, fixation, dehydration, clearing, wax infiltration, embedding, sectioning, staining (using safranin - fast green staining), dewaxing, and sealing. Microscopic observation was performed using an optical microscope (Leica DM2500), and the magnification used was 200×.

## Results

### Gene cloning and sequence analysis

**Figure 1:**
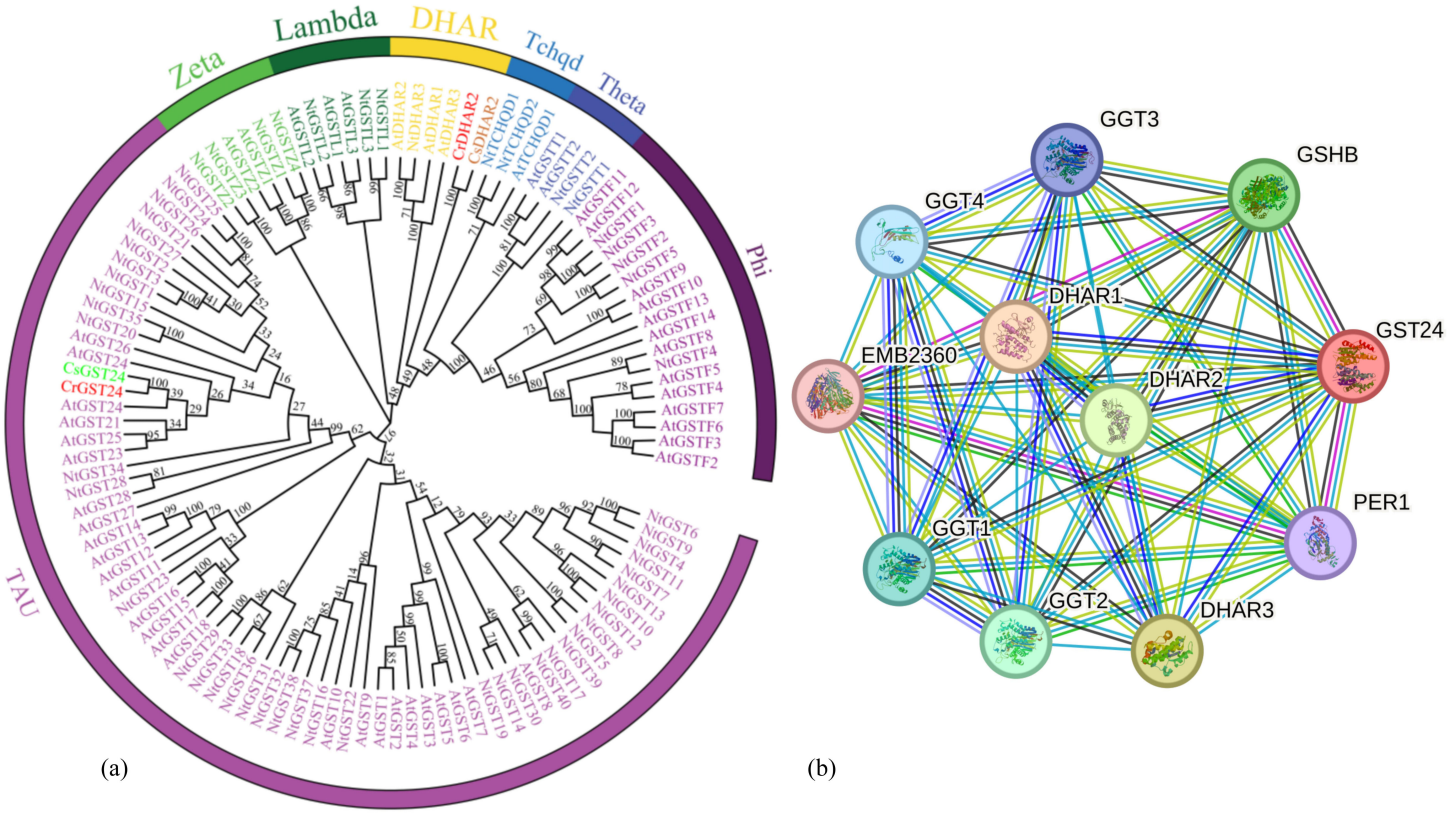
Bioinformatics analysis of the GST from *C reticulata.* Note: **(a)** Phylogenetic tree analysis of GST family members; **(b)** Protein-protein interaction network.

The *GST24* was cloned from callus of *Camellia reticulata*. Multiple sequence alignment and phylogenetic analysis revealed high similarity between this gene and *GST* gene in *Theaceae*, leading to its designation as *CrGST24.* Based on protein homology and gene structure characteristics, the *GST* gene is divided into seven subfamilies: TAU, Zeta, Lambda, DHAR, Tchqd, Theta, and Phi. The *CrGST24* belongs to the TAU, full length is 693 bp and contains an open reading frame encoding 213 amino acids (**Figure 1a**). In order to predict which proteins CrGST24 can interact with, we used the STRING database for analysis, which revealed that CrGST24 may have potential interactions with GSHB and DHAR2 (**Figure 1b**).

### qPCR analysis of *CrGST24* transcription factors

**Figure 2:**
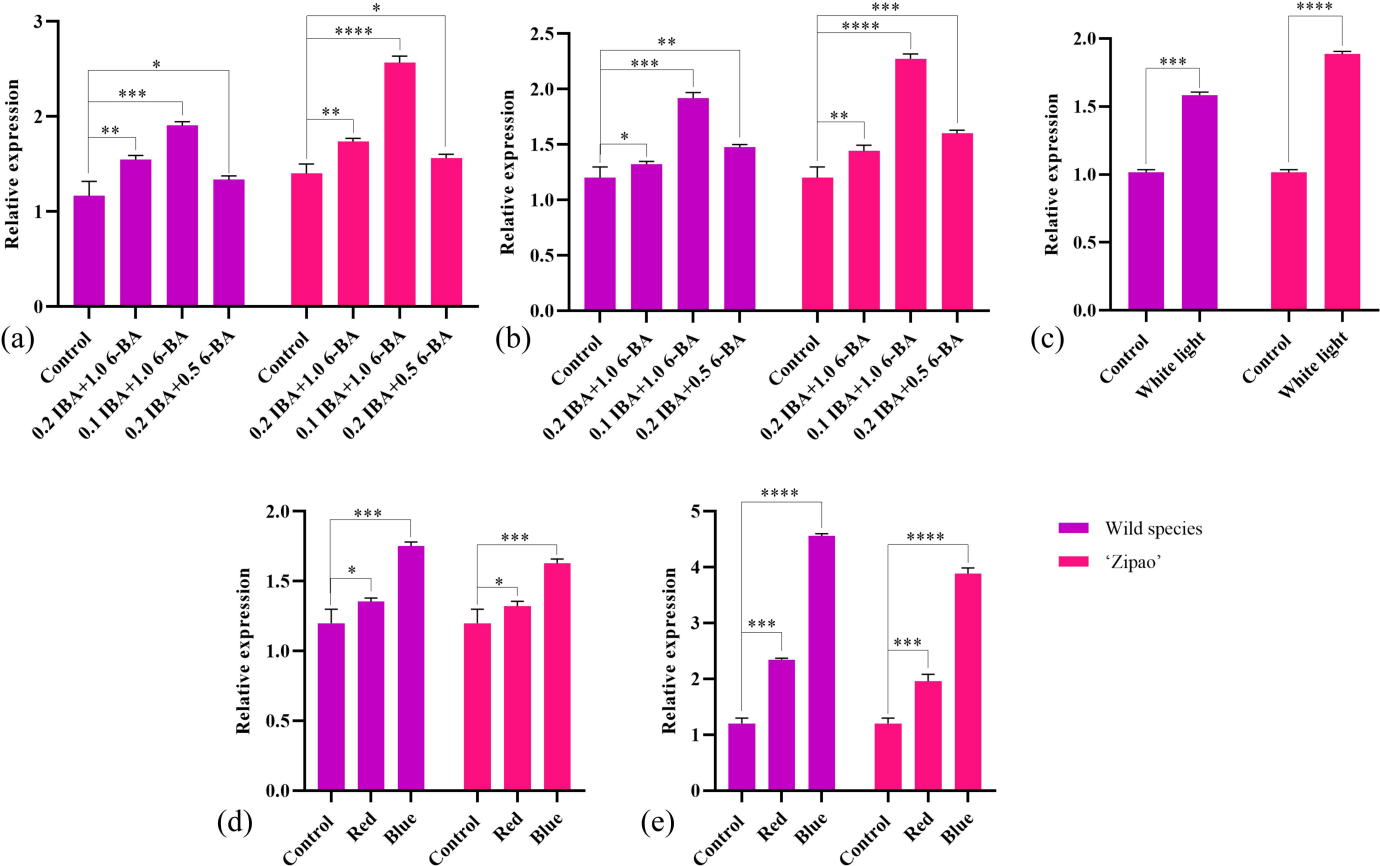
Expression analysis of *CrGST24.* **(a)** *CrGST24* expression under different concentrations of IBA, and 6-BA (2 h); **(b)** *CrGST24* expression under different concentrations of IBA, and 6-BA (1 week); **(c)** *CrGST24* expression under different light conditions (8 h); **(d)** *CrGST24* expression under different light quality (8 h); **(e)** *CrGST24* expression under different light quality (3 d). Note: The bar chart represents the mean of three biological replicates, with error bars showed standard deviations. Asterisks indicate statistical significance based on one-way analysis of variance (*p < 0.05, **p < 0.01, ***p < 0.001, ****p < 0.0001).

The callus of wild species and ‘Zipao’ of *C. reticulata* were placed in medium containing different concentrations of IBA and 6-BA, and treated for 2 h and a week. The expression of *CrGST24* gene was significantly higher than that of the control after 2 h of treatment in medium containing different concentrations of IBA and 6-BA. The expression of *CrGST24* gene in the callus of wild species was 1.55, 1.87, and 1.38 folds higher than that of the control; the expression of *CrGST24* in the callus of ‘Zipao’ was 1.33, 2.03, and 1.23 folds higher than that of the control (**Figure 2a**). The expression of *CrGST24* gene was significantly higher than that of the control after a week of treatment. The expression of *CrGST24* gene in the callus of wild species was 1.30, 1.79 and, 1.33 folds higher than that of the control; the expression of *CrGST24* gene in the callus of ‘Zipao’ was 1.40, 2.27, and 1.57 folds higher than that of the control (**Figure 2b**). The *CrGST24* gene was most highly expressed in the medium of 0.1 mg/L IBA and 1.0 mg/L 6-BA.

The callus of wild species and ‘Zipao’ of *C. reticulata* were placed in YS1 mediun and cultured in dark and white light conditions for 8 h. The dark condition as a control. The expression of *CrGST24* gene was significantly higher than that of control after 8h of treatment under different light conditions. The expression of the *CrGST24* gene in the wild species was 1.53 folds higher than that of the control; the expression of the *CrGST24* gene in the ‘Zipao’ was 1.76 folds higher than that of the control (**Figure 2c**). The *CrGST24* gene responds to light induction.

The callus of wild species and ‘Zipao’ of *C. reticulata* were placed in YS1 medium and cultured in white, red and blue light conditions for 8 h, and 3 d. The white light as a control. The expression of *CrGST24* gene was significantly higher than that of the control after 8 h of treatment in red, and blue light conditions. The expression of *CrGST24* gene in the callus of wild species was 1.31 and 1.75 folds higher than that of the control; the expression of *CrGST24* gene in the callus of ‘Zi Pao’ was 1.28 and 1.63 folds higher than that of the control (**Figure 2d**). The expression of *CrGST24* gene was significantly higher than that of the control after 3 d of treatment. The expression of *CrGST24* gene in the callus of wild species was 2.32 and 4.55 folds higher than that of the control; the expression of *CrGST24* gene in the callus of ‘Zipao’ was 1.87 and 3.85 folds higher than that of the control (**Figure 2e**). The expression of the *CrGST24* gene increased with treatment time, and the *CrGST24* gene was responsive to both red and blue light induction.

### Genetic transformation in tobacco and screening of transgenic tobacco lines

**Figure 3:**
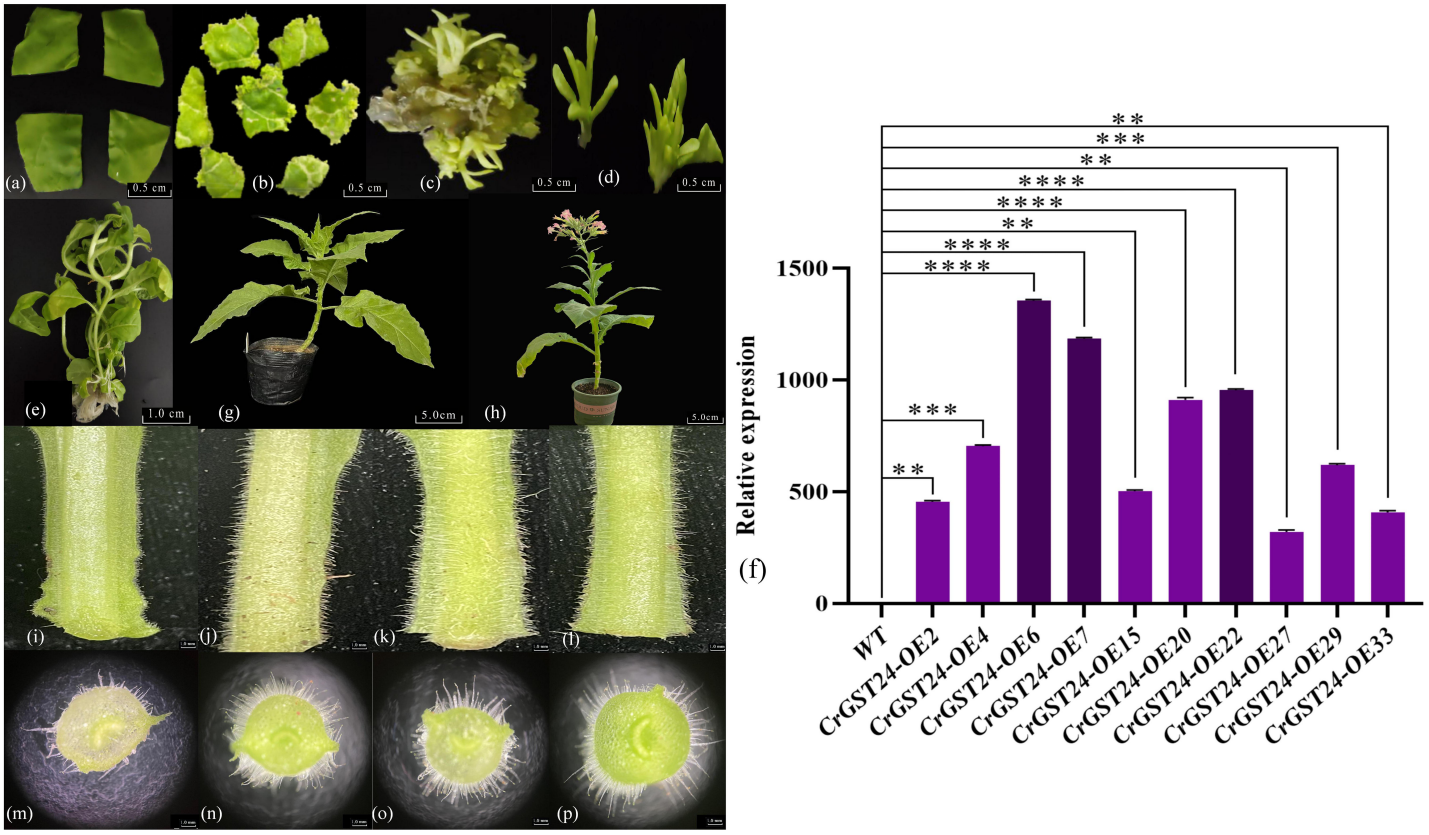
Genetic transformation, positive seedling screening, and phenotypic observation of transgenic tobacco. **(a)** Tobacco leaf discs infected with *Agrobacterium tumefaciens*; **(b)** Induction of resistant buds; **(c)** Expansion of resistant buds; **(d)** Isolation of resistant buds; **(e)** Formation of independent lines; **(f)** Expression analysis of transgenic lines; **(g)** Transgenic tobacco after transplanting; **(h)** Seeds harvested from mature transgenic tobacco for subsequent experiments; **(i, m)** WT tobacco plants; **(j, n)** *CrGST24*-OE6; **(k, o)** *CrGST24*-OE7; **(l, p)** *CrGST24*-OE22. The bar chart represents the mean of three biological replicates, with error bars showing standard deviations. Asterisks indicate statistical significance based on one-way analysis of variance (*p < 0.05, **p < 0.01, ***p < 0.001, ****p < 0.0001).

After *Agrobacterium*-mediated heterologous genetic transformation of tobacco, forty T1 tobacco lines were obtained. Seeds were collected and sown to generate forty T2 lines. Using DNA from the T2 transgenic tobacco as a template, primers targeting the hygromycin transferase gene were designed for PCR, screening out ten positive lines. Through qRT-PCR, three high-expression lines were identified: *CrGST24*-OE6, *CrGST24*-OE7, and *CrGST24*-OE22, with expression levels 1402.37, 1292.74, and 971.02 folds higher than that in WT (**Figures 3a-h**).

### Phenotype observation of the *CrGST24* transgenic lines

The length and density of stem trichomes in transgenic tobacco were superior to those in WT (**Figures 3i-l**). Electron microscopy of freehand sections revealed that WT had approximately 35-40 trichomes per field of view (20x), with lengths ranging from 1.02 to 1.51 mm and an average length of 1.32 mm. In contrast, transgenic tobacco exhibited 75-80 trichomes, with lengths ranging from 2.11 to 3.64 mm and an average length of about 3.13 mm. This resulted in trichome lengths and densities that were 2.37 folds higher than those of the WT (**Figures 3m-p**).

### Observations on *in vitro* regeneration of transgenic tobacco

**Figure 4:**
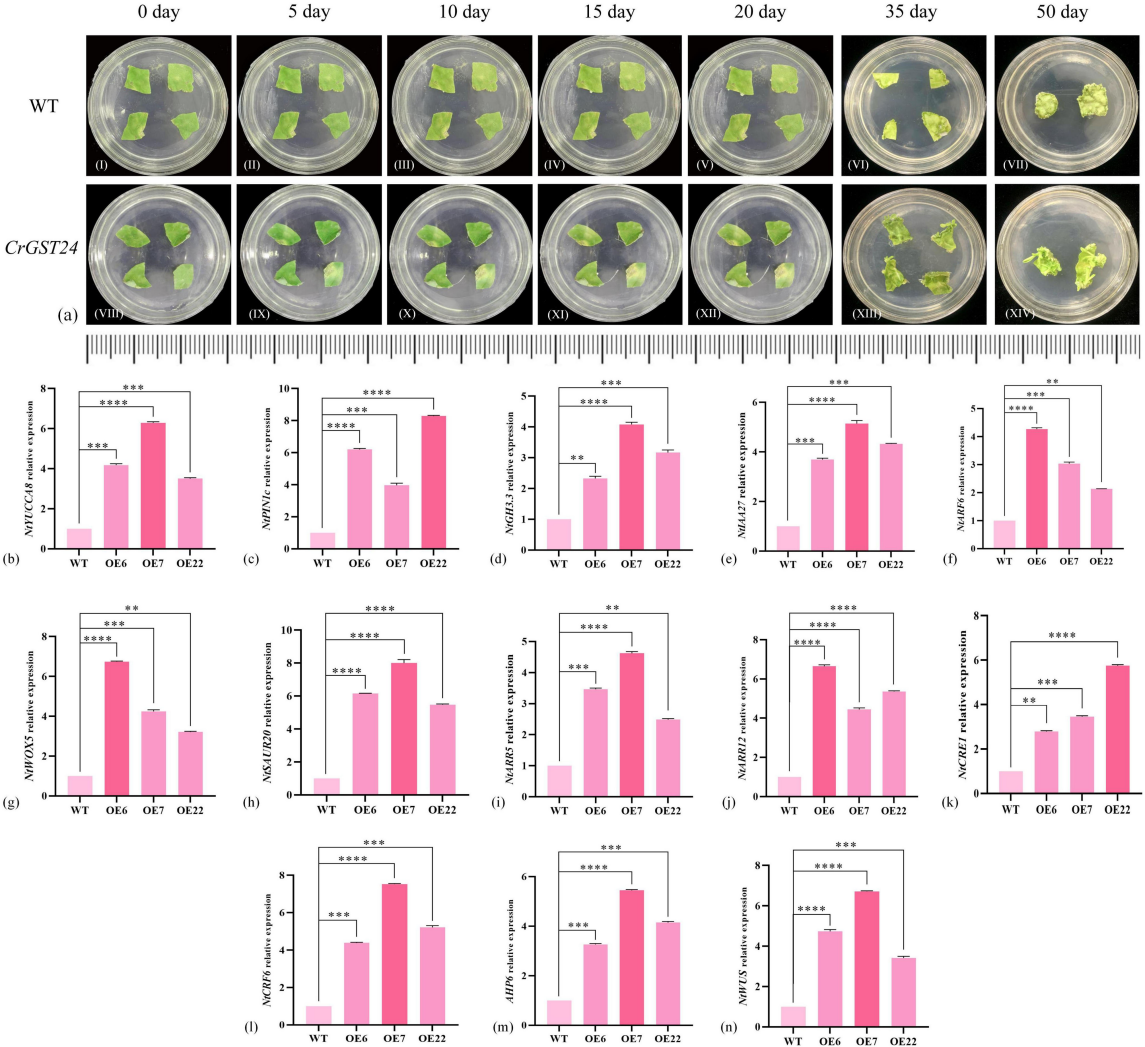
Observations on *in vitro* regeneration of *CrGST24* transgenic tobacco and expression of auxin and cytokinin-related genes in *CrGST24* transgenic tobacco. **(a)** *in vitro* regeneration **(b-h)** auxin-related genes; **(i-n)** cytokinin-related genes. The bar chart represents the mean of three biological replicates, with error bars showing standard deviations. Asterisks indicate statistical significance based on one-way analysis of variance (*p < 0.05, **p < 0.01, ***p < 0.001, ****p < 0.0001).

The leaves of WT and transgenic tobacco were cut into uniform sizes, and then placed in normal MS medium to observe the growth. The WT leaves started to differentiate callus on the 10th day and as the treatment time increased, more callus were differentiated from the WT and by the 20th day, four callus were differentiated from the WT leaves. Leaves of *CrGST24* transgenic tobacco showed signs of differentiation of adventitious roots and callus at the 5th day, with adventitious roots of about 0.1 mm in length; on the 10th day adventitious roots continued to grow and were about 0.2-0.5 mm in length; The length of the adventitious roots was about 0.4-0.7 mm on the 15th day; the length of the adventitious roots was about 0.5-0.8 mm on the 20th day. On the 35th day, both WT and transgenic tobacco plants produced adventitious buds. Compared with the WT, the adventitious buds produced by the transgenic tobacco were more obvious. On the 50th day, the adventitious buds of WT gradually grew larger, while those of transgenic tobacco had grown into independent plants (**Figure 4a**).

### Analysis of auxin and cytokinin-related gene expression in *CrGST24* transgenic tobacco

Based on the leaf regeneration of transgenic tobacco *in vitro*, we found auxin and cytokinin-related genes in tobacco at the NCBI online website, and analysed the expression of these genes in transgenic tobacco by q-PCR. The expression of auxin-related genes in transgenic tobacco were all significantly increased and significantly higher than the WT. The expression of *NtYUCCA8* in the three transgenic tobacco lines were 3.98, 6.07, and 3.54 folds higher than the WT (**Figure 4b**); *NtPIN1c* were 6.11, 3.87, and 7.97 folds higher than the WT (**Figure 4c**); *NtGH3.3* were 2.32, 4.03, and 3.12 folds higher than the WT (**Figure 4d**); *NtIAA27* were 3.68, 4.97, and 4.33 folds higher than the WT (**Figure 4e**); *NtARF6* were 4.18, 2.89, and 1.93 folds higher than the WT (**Figure 4f**); *NtWOX5* were 6.36, 4.02, and 3.47 folds higher than the WT (**Figure 4g**); *NtSAUR20* were 5.87, 7.92, and 5.71 folds higher than the WT (**Figure 4h**).

The expression of cytokinin-related genes were significantly increased in transgenic tobacco and was significantly higher than the WT. *NtARR5* were 3.24, 4.27, and 2.18 folds higher than the WT (**Figure 4i**); *NtARR12* were 6.22, 4.12, and 5.28 folds higher than the WT (**Figure 4j**); *NtCRE1* were 2.48, 3.67, and 5.69 folds higher than the WT (**Figure 4k**); *NtCRF6* were 3.76, 5.71, and 3.98 folds higher than the WT (**Figure 4l**); *NtAHP6* were 4.07, 7.39, and 5.24 folds higher than the WT (**Figure 4m**); *NtWUS* were 4.32, 6.26, and 3.77 folds higher than the WT (**Figure 4n**). The *CrGST24* gene promoted the expression of both auxin and cytokinin-related genes.

### Expression analysis of auxin and cytokinin-related genes in the callus of *C. reticulata*

**Figure 5:**
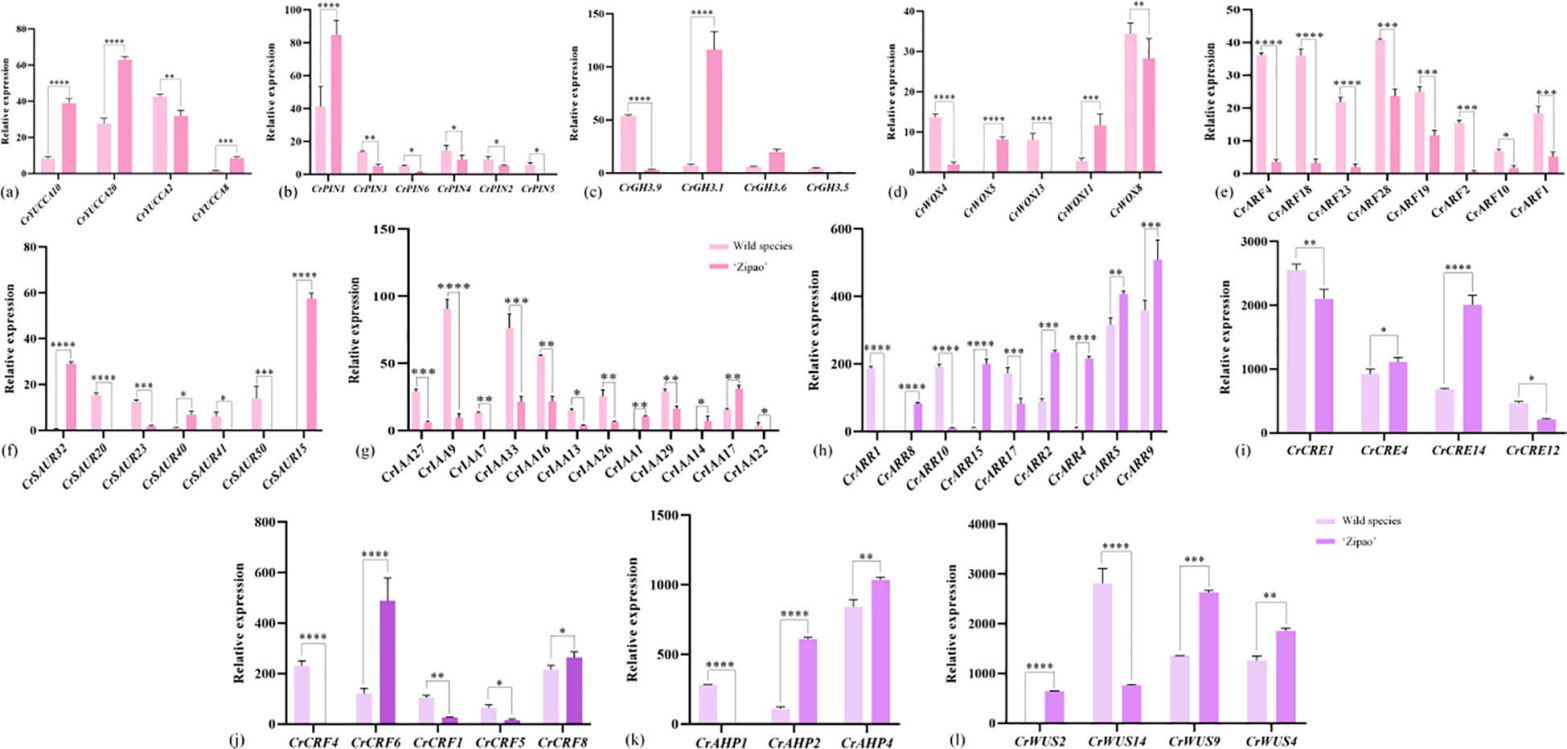
Expression of auxin and cytokinin-related genes in *C. reticulata* callus. **(a-g)** Auxin-related genes; **(h-l)** Cytokinin-related genes. The bar chart represents the mean of three biological replicates, with error bars showing standard deviations. Asterisks indicate statistical significance based on one-way analysis of variance (*p < 0.05, **p < 0.01, ***p < 0.001, ****p < 0.0001).

A total of 12 auxin and cytokinin-related genes were screened in the transcriptome data of *C. reticulata* callus. There are seven auxin-related genes, including: *CrYUCCA*, *CrPIN*, *CrGH3*, *CrWOX*, *CrARF*, *CrSAUR* and *CrIAA*. The most significant differences in the expression of *CrYUCCA2*, *CrPIN3*, *CrGH3.9*, *CrWOX13*, *CrARF4*, *CrSAUR20* and *CrIAA9* were found in the callus of the wild species, which were higher than ‘Zipao’ by 1.36, 2.41, 4.17, 8.25, 11.67, 17.24 and 7.73 folds; the most significant differences in the expression of *CrYUCCA10*, *CrPIN1*, *CrGH3.1*, *CrWOX5*, *CrSAUR15* and *CrIAA17* were found in the callus of ‘Zipao’, which were 4.88, 2.05, 10.12, 8.85, 52.51, and 1.43 folds higher than that of the wild species (**Figures 5a-g**).

There were five cytokinin-related genes, including *CrARR*, *CrCRE*, *CrCRF*, *CrAHP*, and *CrWUS.* The most significant differences in the expression of *CrARR1*, *CrCRE1*, *CrCRF4*, *CrAHP1*, and *CrWUS14* were found in the callus of the wild species, which were 188.95, 1.25, 205.32, 237.02, and 3.11 folds higher than that of ‘Zipao’. The most significant differences in the expression of *CrARR4*, *CrCRE14*, *CrCRF6*, *CrAHP2*, and *CrWUS9* were found in ‘Zipao’ callus, which were 201.14, 2.51, 3.58, 5.67, and 2.48 folds higher than that of the wild species (**Figures 5h-l**).

### Determination of auxin, cytokinin, ROS, GSH and AsA contents in *CrGST24* transgenic tobacco

**Figure 6:**
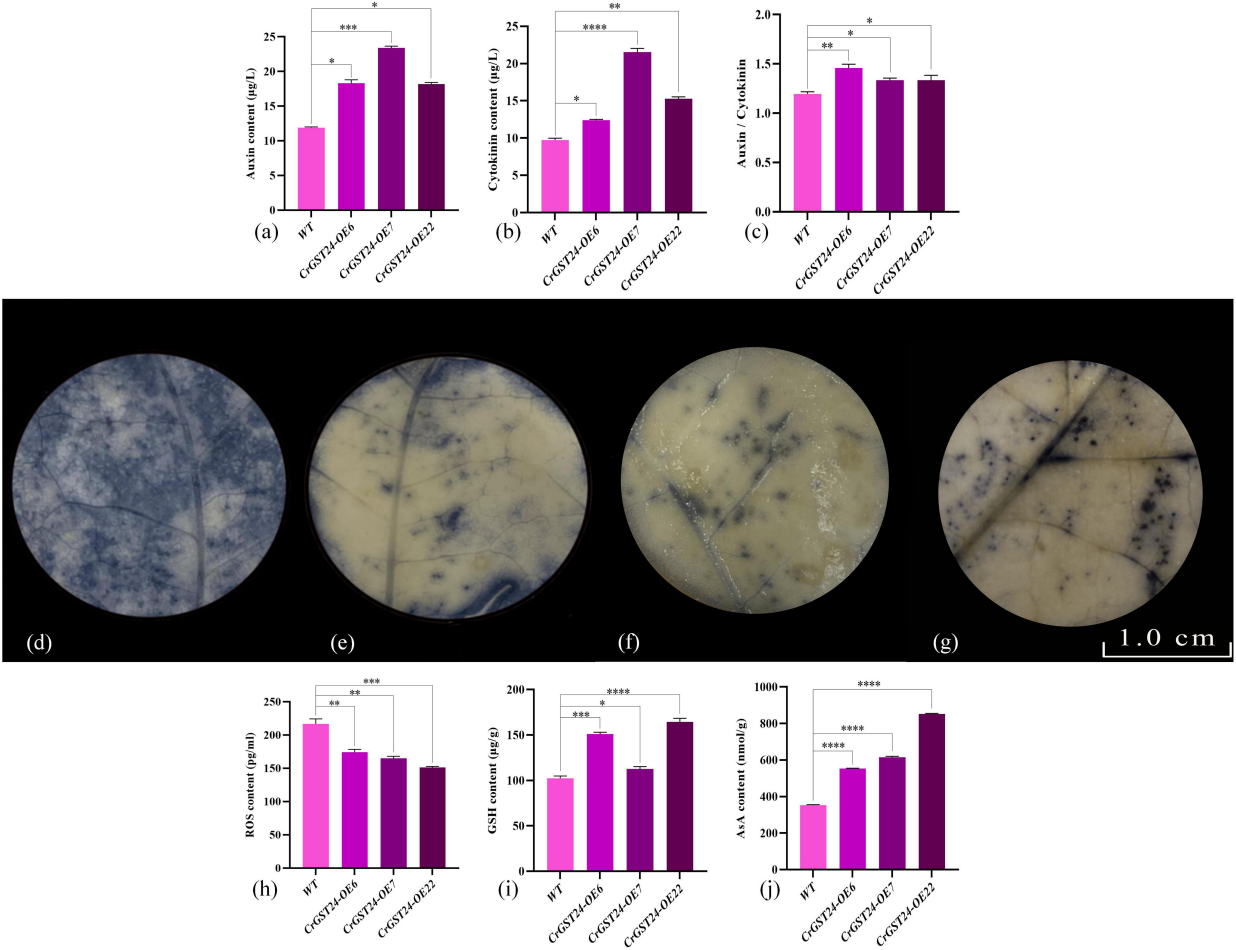
Expression of auxin, cytokinin, ROS, GSH, and AsA contents in *CrGST24* transgenic tobacco. Note: **(a)** Auxin content; **(b)** Cytokinin content; **(c)** Auxin/cytokinin content; **(d)** WT; **(e)** *CrGST24*-OE6; **(f)** *CrGST24*-OE7; **(g)** *CrGST24*-OE22; **(h)** ROS content; **(i)** GSH content; **(j)** AsA content; **(d-g)** the blue part is the area of ROS accumulation. The bar chart represents the mean of three biological replicates, with error bars showing standard deviations. Asterisks indicate statistical significance based on one-way analysis of variance (*p < 0.05, **p < 0.01, ***p < 0.001, ****p < 0.0001).

When the auxin and cytokinin contents within transgenic tobacco was examined, the auxin contents in transgenic tobacco were significantly higher than that in WT, which were 1.55, 2.09, and 1.47 folds higher than that in WT (**Figure 6a**); cytokinin contents in transgenic tobacco were significantly higher than that in WT, which were 1.13, 2.16, and 1.54 folds higher than that in WT (**Figure 6b**). The contents of auxin were significantly higher than that of cytokinin in transgenic tobacco, and the ratios of auxin and cytokinin contents in WT and transgenic tobacco were analysed, and the ratios of auxin and cytokinin contents in *CrGST24* transgenic tobacco were significantly higher than those in WT, which were 1.24, 1.13, and 1.15 folds higher than those in WT (**Figure 6c**).

NBT staining followed by decolourisation of WT and transgenic tobacco revealed that the area of ROS was significantly larger in WT than in transgenic tobacco (**Figures 6d-g**). The detection of ROS contents in both WT and transgenic tobacco revealed that the ROS contents of transgenic tobacco were significantly lower than that of WT, 1.15, 1.07 and 1.31 folds lower than that of WT (**Figure 6h**); the GSH contents of transgenic tobacco were all significantly higher than those of WT, 1.27, 1.42 and 1.35 folds higher than those of WT (**Figure 6i**); the AsA content of transgenic tobacco were all significant higher than those of WT, 6.09, 4.47, and 2.92 folds higher than those of WT (**Figure 6j**).

### The interaction between *CrGST24* and *CrDHAR2*, CrGSHB

**Figure 7:**
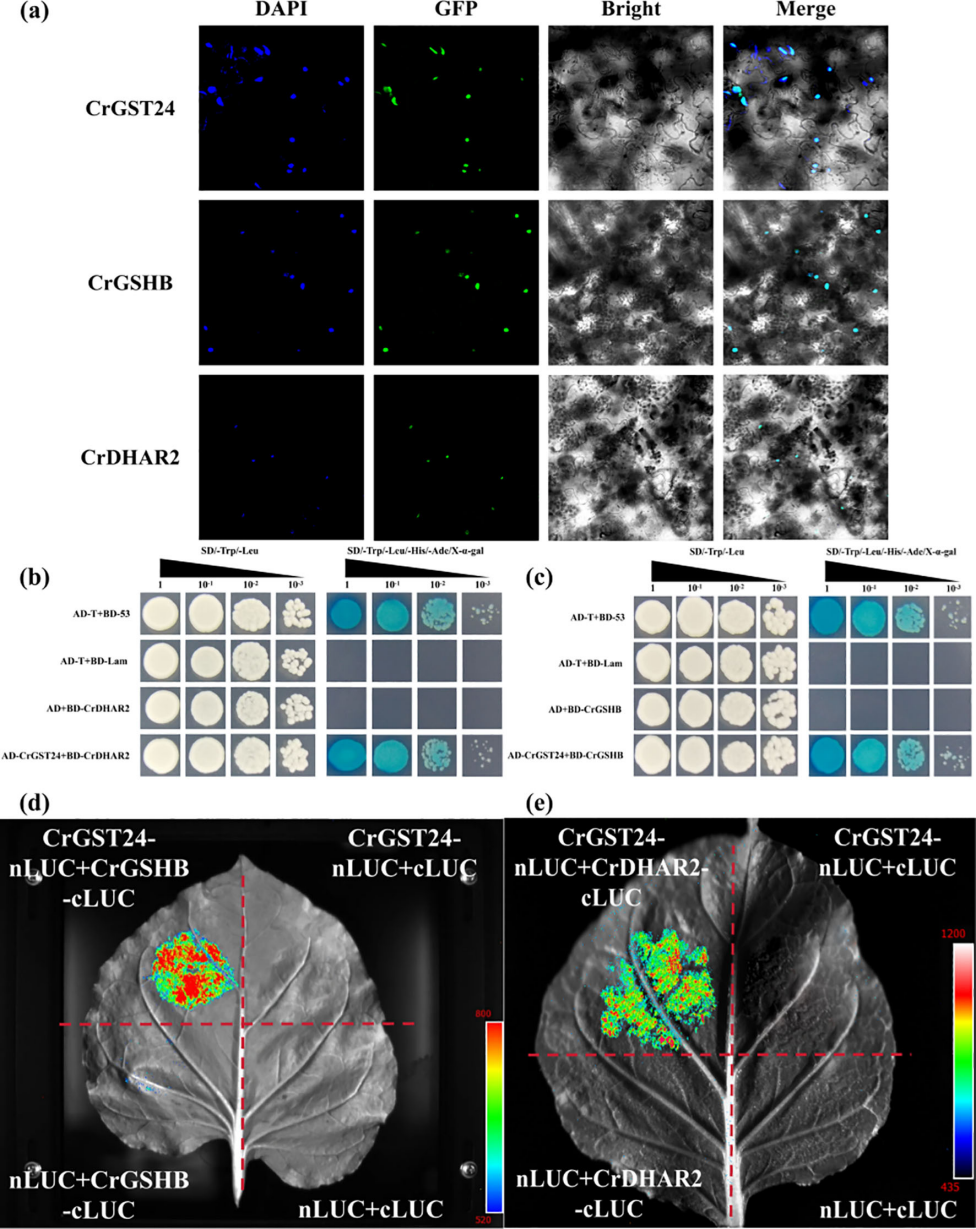
*CrGST24* interact with *CrGSHB* and *CrDHAR2.* **(a)** Subcellular localization assay; **(b, c)** Y2H assay; **(d, e)** LUC assay.

The subcellular localization results showed that *CrGST24*, *CrGSHB*, and *CrDHAR2* are all located in the nucleus (**Figure 7a**). We screened key genes involved in AsA and GSH synthesis by yeast two-hybrid assay, and showed that the *CrGST24* gene could interact with *CrGSHB* and *CrDHAR2* (**Figures 7b, c**). The LUC experiment further confirmed that *CrGST24* interacted with *CrGSHB* and *CrDHAR2* (**Figures 7d, e**).

### Role of different concentrations of IBA, 6-BA and different light qualities in *C. reticulata* callus culture

**Figure 8:**
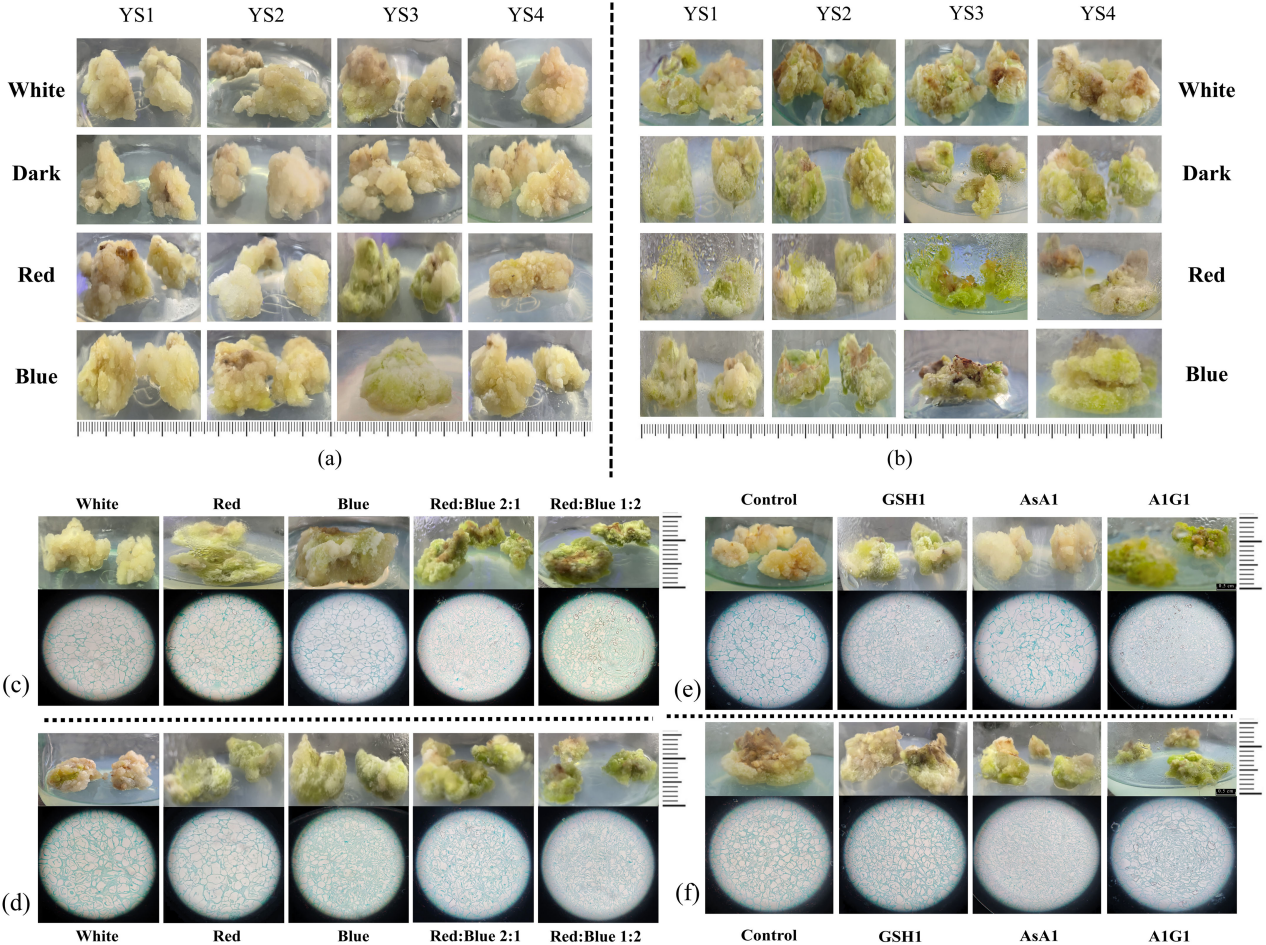
Growth status of callus from *C. reticulata.* **(a, b)** Callus in medium contaning different concentrations of IBA, 6-BA, and different light quality treatments; **(c, d)** Callus under red light, blue light, and red-blue light; **(e, f)** Callus under GSH and AsA treatments. **(a, c, e)** ‘Zipao’; **(b, d, f)** wild species.

The callus of ‘Zipao’ and wild species were placed on YS1, YS2, YS3 and YS4 callus medium containing different concentrations of IBA and 6-BA, and they were incubated with white light, darkness, red light, or blue light for 30 d to observe the growth of the callus. In comparison with the white loose tissue structure before treatment and other different treatments, ‘Zipao’ callus in YS3 medium and treated with red light or blue light respectively had obvious changes in colour from white to green, and the tissue structure changed from loose to denser, and the newly differentiated callus also showed obvious green colour (**Figure 8a**). The wild species grew better in YS3 medium and under red or blue light treatments, with denser tissue structure and subsequent newly differentiated healing tissues that were also distinctly green in colour (**Figure 8b**). The above results indicated that YS3 medium, as well as red or blue light were good for the *C. reticulata* callus growth.

### Analysis of the growth of *C. reticulata* callus under red and blue light environments

Callus of ‘Zipao’ and wild species were inoculated on YS3 medium, or under four different light quality treatments, including red, blue, red-blue ratio of 2:1, and blue-red ratio of 2:1 for one month with white light as the control. Under the four different light quality treatments, the callus of ‘Zipao’ changed significantly from the original white loose tissue to the green firm healing tissue, and the growth condition was better under blue-red ratio of 2:1. The paraffin sections showed that the cells of callus were compact under the red or blue light treatment compared to the white light; the cells were tightly packed under red-blue ratio of 2:1 or blue-red ratio of 2:1, and compared with red-blue ratio of 2:1, the cells were much more tightly under blue-red ratio of 2:1 (**Figure 8c**).

There was no significant difference in the morphological characteristics of wild species callus under red or blue light compared with white light, but the morphological characteristics changed significantly after red-blue ratio of 2:1 and blue-red ratio of 2:1, with the colour changing from dark green to bright green. The paraffin sections showed that the degree of cell tightness under red or blue light was not significantly different from that after white light treatment; cells treated under red-blue ratio of 2:1 or blue-red ratio of 2:1 were tight, and the tightness of the cells was made higher under the blue-red ratio of 2:1 treatment (**Figure 8d**).

### Role of GSH and AsA in the induction of adventitious shoots in the callus of *C. reticulata* “Zipao” and wild species

The wild species callus treated with GSH1 changed from light green to green and became tighter, and the cells were more tight according to the paraffin sections; the wild species callus treated with AsA1 did not change significantly; the best growth of wild species callus were observed after A1G1 treatment, with bright green and tight cells (**Figure 8e**).

The ‘Zipao’ callus treated with GSH1 changed from white to green and became tighter, and the cells of ‘Zipao’ callus treated with GSH1 were more tight according to the paraffin sections; there were no significant changes in the external morphology and internal structure of ‘Zipao’ callus treated with AsA1 compared to those before treatment; ‘Zipao’ callus treated with A1G1 showed the best growth, in terms of external colour the ‘Zipao’ callus treated with A1G1 were bright green in colour compared to the pre-treatment and GSH1 treatment, and in terms of cellular structure the cultivar healing cells were highly aggregated in the A1G1 treatment (**Figure 8f**).

## Discussion

In the previous study, when the auxin was added exogenously or synthesised endogenously, it will bind to the promoter region of the downstream *GST* gene through a series of pathways and activate the expression of the *GST* gene, and when the *GST* gene is overexpressed, it will promote the synthesis of GSH, which will form a cycle with AsA, and thus inhibit or remove ROS (**Supplementary Figure 2**). The conclusions obtained in this study were consistent with previous studies that *CrGST24* was able to promote the synthesis of endogenous auxin and cytokinins in transgenic tobacco, and that the content of GSH and AsA was significantly higher and the content of ROS was significantly lower in *CrGST24* transgenic tobacco than in WT tobacco.

### *GST* genes and phytohormones in plant growth

In this study, we found that *CrGST24* could promote the synthesis of endogenous auxin and cytokinin. It also caused changes in the expression of auxin and cytokinin - related transcription factors such as *YUCCA*, *ARF*, *GH3*, *WOX*, *WUS*, *CRF*, *AHP*, *CRE*, and *ARR*. Overexpression of the *YUCCA* could significantly increase the auxin content in plants, thereby affecting their growth and development (Cao et al., 2019). In *Arabidopsis thaliana*, knocking out the *YUCCA* resulted in diminished adventitious bud formation capacity, whereas overexpression of the *YUCCA* enhanced the ability of adventitious bud differentiation (Miguel et al., 2020). In rice, overexpression of *OsWOX3A* led to significantly upregulated expression of *PIN1*. The resulting change in auxin transport direction caused by *PIN1* led to local concentration gradients. This process activated the expression of *YUCCA* genes through feedback regulation, thereby promoting the synthesis of endogenous auxin (Sung and Paek, 2016). The *WOX5* gene interacted with auxin and cytokinin signaling pathways. Specifically, by regulating the *ARR* genes, it affected the sensitivity to cytokinins, thereby promoting the differentiation of adventitious buds from callus (Zhai and Xu, 2021). In plant biology, the *ARF* transcription factor activated *GH3* and *SAUR* genes, thereby promoted the formation of IAA conjugates and increased the levels of endogenous auxin (Dong et al., 2024). The cytokinin signal was transmitted through the AHK-AHP-ARR cascade (To and Kieber, 2008). Once activated, specific types of *ARR* directly bound to the promoter of *CRF* and upregulated their expression. Meanwhile, *AHP* interacted with *CRF*, promoting their nuclear accumulation. This formed a coordinated regulatory module between *CRF* and *ARR*, which together activated downstream cytokinin - response genes, thereby promoted cytokinin synthesis (Cutcliffe et al., 2011). Previous studies have found that the transcription factors *YUCCA*, *PIN*, *WOX*, *SAUR*, *ARF*, *ARR*, *AHP*, and *CRF* have extremely important functions in promoting the synthesis of auxin and cytokinin. In the transcriptome data of *C. reticulata* callus, the auxin and cytokinin-related transcription factors were also found. Combined with the results of this study, it is hypothesized that these transcription factors may bind to the promoter of the *CrGST24* gene and activate its expression. Along with exogenous IBA and 6-BA, as well as red - blue light induction, this process promotes the synthesis of endogenous auxin and cytokinin.

### The role of GSH and AsA in adventitious bud differentiation

GSH and AsA have extremely important roles in plant growth, especially in the scavenging of ROS (Hasanuzzaman et al., 2020). In this study we found that the *CrGST24* gene promoted the synthesis of GSH and AsA in transgenic tobacco, and that the content of glutathione and ascorbic acid in *CrGST24* transgenic tobacco was significantly lower than that in WT tobacco. Better growth of *C. reticulata* callus treated with GSH and AsA. GSH has an extremely important role in the differentiation of adventitious buds (Elaziem and Ahmed, 2020). In the induction of adventitious buds of *Phoenix dactylifera*, the addition of 30 mg/L GSH to the proliferation medium significantly promoted the differentiation of adventitious buds. After four generations of subculture, the number of adventitious buds significantly increased in the medium containing 30 mg/L GSH and 0.5 mg/L TDZ (Elaziem and Ahmed, 2020). GSH enhanced plant adventitious shoot differentiation by regulated intracellular iron homeostasis, participated in signal transduction pathways and improved the redox environment of the medium (Ranjana et al., 2021). Adding a certain amount of GSH to the culture medium reduced the secretion of phenolic substances, prevented browning of plant materials, and improved the induction efficiency of adventitious buds in bananas (Skogerboe and Jenderek, 2022). Glutathione is an important antioxidant in plant cells, and the dynamic balance between its reduced (GSH) and oxidised (GSSG) states (GSH/GSSG) directly affects the redox state of cells. During adventitious bud differentiation, accumulation of reactive oxygen species (ROS) inhibited cell dedifferentiation or redifferentiation. Glutathione protected DNA, proteins and membrane systems from oxidative damage by scavenging ROS, thus provided a stable microenvironment for callus formation and bud primordial differentiation (Müller et al., 2020). AsA plays an important role in cell division and growth, and AsA has an extremely important role in the growth of plant roots and adventitious shoots (Akram et al., 2017). When ascorbate peroxidase (APX1) was knocked out in oilseed rape, the differentiation rate of adventitious shoots was drastically reduced (Liang et al., 2024). Addition of AsA to the culture medium significantly increased the efficiency of inducing adventitious shoots in sorghum (Baskaran and Jayabalan, 2005). After blue light treatment of wheat seedlings, the contents of POD, SOD, GSH and AsA in wheat leaves significantly increased, while the ROS content markedly decreased. This indicated that blue light treatment could enhance the antioxidant capacity of wheat seedlings (Li et al., 2022). *GST* genes were closely related to GSH and AsA synthesis. In this study we found that *CrGST24* promoted GSH and AsA synthesis in transgenic tobacco. The callus treated with GSH and AsA grew better. Excessive ROS could severely inhibit dedifferentiation and redifferentiation of plant callus. We hypothesised that when the GSH and AsA contents were elevated in *C. reticulata* callus then excessive ROS would be scavenged; exogenous addition of GSH and AsA, on the other hand, inhibits the secretion of phenolics and keeps the browning of healing tissues in culture under control. When the GSH and AsA contents in the healing tissue increased significantly, and at the same time GSH and AsA were added exogenously, a double safeguard was formed, which promoted the differentiation of adventitious shoots in the *C. reticulata* callus. The synthesis of GSH and AsA is a complex mechanism that requires further study.

### The co-action of *GST* genes with other genes in plant growth

*GST* gene enhances plant resistance to biotic and abiotic stresses by scavenging excess ROS. In this study, overexpression of the *CrGST24* gene promoted the synthesis of GSH and AsA in transgenic tobacco, and the ROS content in transgenic tobacco was significantly lower than that in WT tobacco. We found that the *CrGST24* gene could interact with *CrGSHB*, a key gene for GSH synthesis, and *CrDHAR2*, a key gene for AsA synthesis, by Y2H assay. *GSHB* genes have an important role in plant resistance to adversity stress (Anjuman et al., 2025). Numerous studies have shown that *DHAR* and *GSHB* genes have extremely important roles in plant resistance to biotic and abiotic stresses, and in scavenging ROS. *DHAR* has an important role in the synthesis of AsA, *DHAR* maintains intracellular AsA levels in the reduced state by reducing dehydroascorbic acid (DHA) to AsA. *DHAR* protected cells from oxidative damage by promoted AsA synthesis and scavenged intracellular ROS (Petrova and Smith, 2015). When the *DHAR* gene was knocked out, the content of AsA was significantly reduced and the antioxidant capacity of the plant was reduced (Terai et al., 2020). In rice, overexpression of the *DHAR* gene increased rice resistance to salt stress by reducing H_2_O_2_ levels (Kim et al., 2017). *GSHB* is a glutathione synthetase that adds glycine to γ-glutamylcysteine to eventually produce GSH. In *Brassica campestris*, overexpression of the *GSHB* gene significantly promoted GSH synthesis, which improved antioxidant capacity and stress tolerance in *Brassica campestris* (Yan et al., 2013). *GSHB* combines γ-glutamylcysteine (γ-Glu-Cys), which is produced by catalyzing glutamate, cysteine, and γ-glutamyltransferase (GshA), with glycine to ultimately form glutathione (GSH). This process is an important mechanism for cellular antioxidation and maintenance of redox homeostasis (Copley and Dhillon, 2002). In cyanobacteria, GSH cannot be synthesized when the *GSHB* gene was knocked out (Okumura et al., 1997). Overexpression of the *GSHB* gene in Arabidopsis promoted GSH synthesis and also improved tolerance to Cd stress in Arabidopsis (Kumar and Chattopadhyay, 2018). In AsA-GSH cycle, *DHAR* used reduced glutathione (GSH) as a hydrogen donor to catalyzed DHA production of AsA. Under oxidative stress, the AsA synthesis pathway is limited, and *DHAR* compensated for the synthesis deficit by regenerated AsA. For example, under drought conditions, elevated *DHAR* activity promoted AsA synthesis (Hasanuzzaman et al., 2020). In transgenic tobacco, overexpression of DHAR gene increased AsA content by 2-4 folds (Wang et al., 2010). Based on the previous studies and the results of this research, we speculated that *CrGST24* could utilize GSH as a cofactor to convert harmful ROS into harmless substances. *CrGSHB* enhanced the efficiency of the entire antioxidant system by synthesizing more GSH to provide sufficient substrates for *CrGST24*. When the *CrGST24* gene was overexpressed, the expression of the *CrGSHB* gene may be promoted, thereby increased the synthesis of GSHB enzyme. More GSHB enzymes could catalyze more γ-Glu-Cys and Gly to synthesize GSH, thereby led to an increase in GSH content. When the *CrGST24* gene was overexpressed, it may promote the synthesis of GSH or reduce the consumption of GSH through some mechanism, thereby providing more GSH as a substrate for *CrDHAR2*. Sufficient supply of GSH is conducive to *CrDHAR2* catalyzing the reduction reaction of DHA more effectively, thereby increasing the content of AsA. The interaction between *CrGST24* gene and *CrDHAR2* gene may activate the entire AsA-GSH cycle system. Besides *CrDHAR2*, the activities of other related enzymes in the cycle (such as APX, GR, etc.) may also be coordinately regulated, thereby promoting the synthesis and regeneration of AsA. When the *CrGST24* gene interacted with the *CrGSHB* and *CrDHAR2* genes, it activated the entire AsA-GSH cycle system, regulated the expression of some key genes and thereby promoted the synthesis of GSH and AsA.

## Conclusion

**Figure 9:**
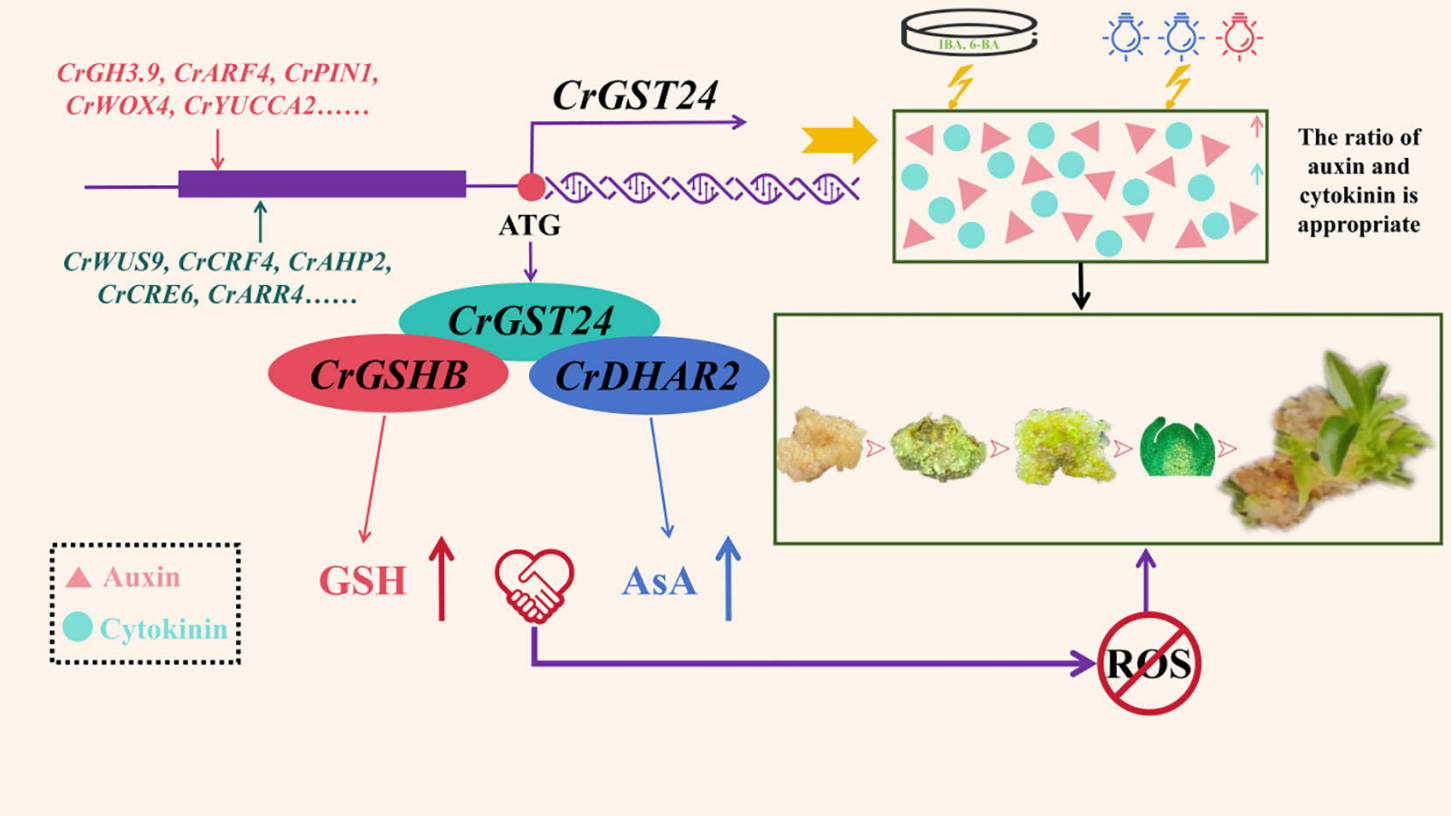
The mechanism of *CrGST24* gene in *C. reticulata* promoting the formation of adventitious buds from callus differentiation.

In this study, we found that the *CrGST24* gene could promote the synthesis of endogenous auxin and cytokinin and influence their ratio, which was regulated by some auxin and cytokinin - related transcription factors, including *CrWUS9*, *CrCRF4*, *CrAHP2*, *CrCRE6*, *CrGH3.9*, *CrARF4*, *CrPIN1*, and *CrWOX4*. The elevation of the levels of endogenous auxin and cytokinin in the callus of *C.reticulata* through the treatment of exogenous IBA, 6-BA and red-blue light could increase the differentiation rate of adventitious buds, proving the above view. Moreover, the *CrGST24* gene interacted with the *CrGSHB* and *CrDHAR2* genes, thereby facilitating the synthesis of GSH and AsA and scavenging ROS. Thus, under the joint influence of the ratio of auxin and cytokinin and the scavenging ROS regulated by *CrGST24*, the adventitious bud differentiation was facilitated (**Figure 9**).

## Data Availability

The original contributions presented in the study are included in the article/**Supplementary Material**. Further inquiries can be directed to the corresponding author.

## Supplementary material

Supplementary Figure 1 Callus of *Camellia reticulata* used in the experiment. **(a)** ‘Zipao’; **(b)** Wild species

Supplementary Figure 2 Schematic diagram of auxin and glutathione synthesis (Moones., 2005).

Supplementary Table 1 The culture medium required for the research.
