# Supplementary figures and images for: Glutathione S-transferase CrGST24 in the differentiation of adventitious buds from Camellia reticulata callus
Source: Front Plant Sci. 2025 Aug 15;16:1641401. doi: 10.3389/fpls.2025.1641401 (PMC12395571; doi:10.3389/fpls.2025.1641401)

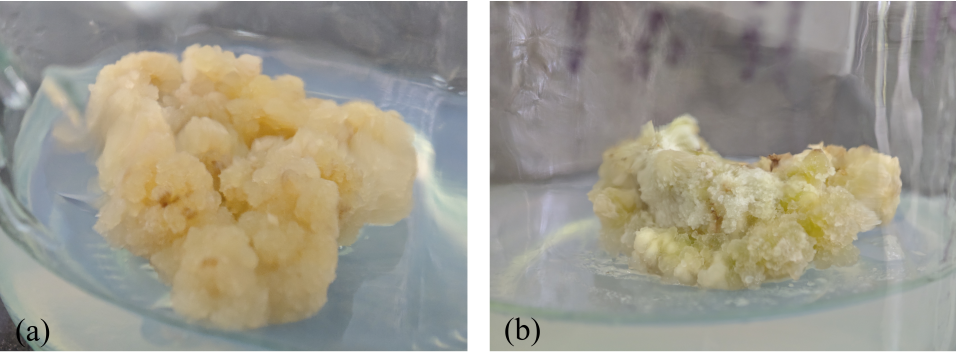

Supplement: Supplementary Figure 1 — Callus of Camellia reticulata used in the experiment. (a) ‘Zipao’; (b) Wild species [file Image1.png]

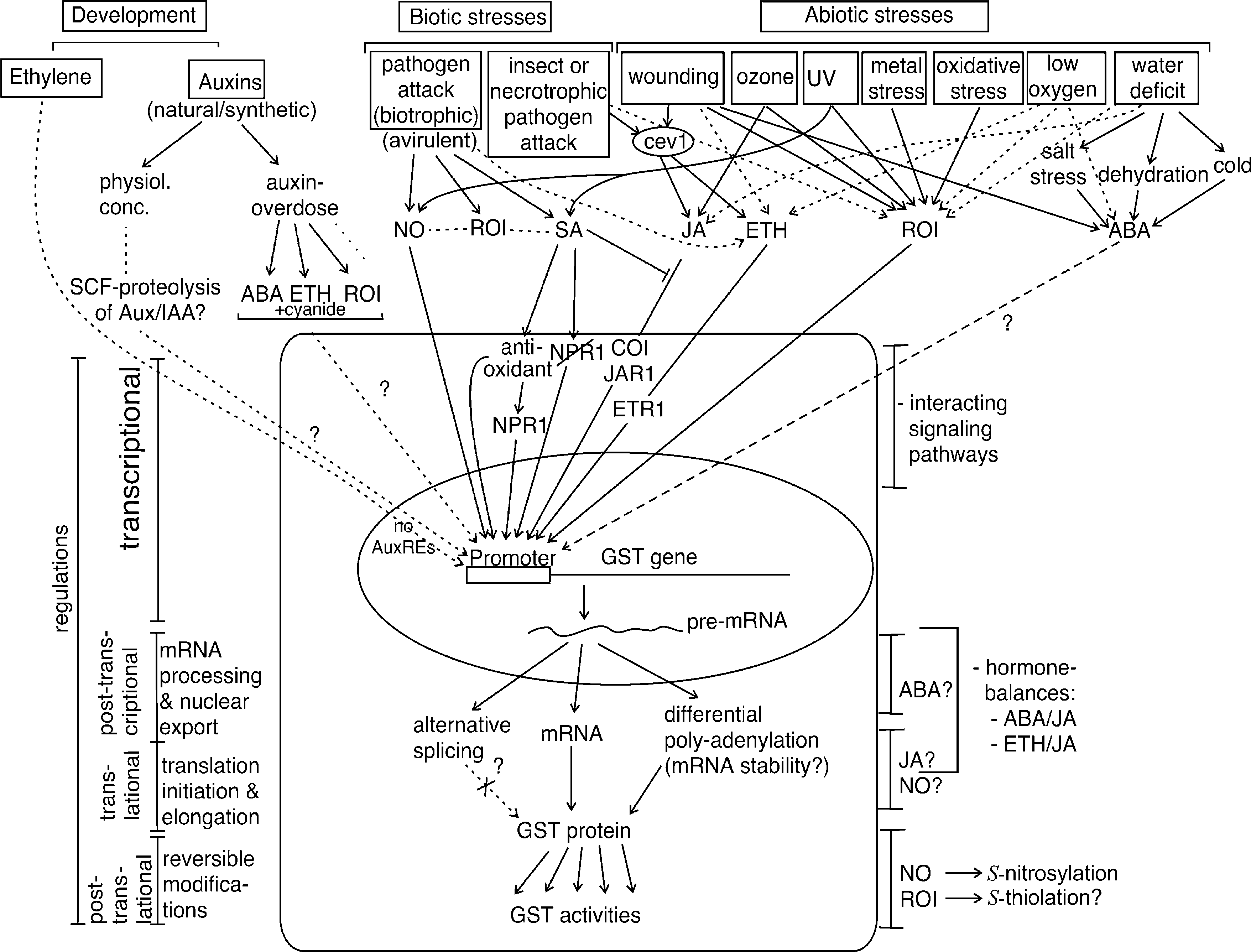

Supplement: Supplementary Figure 2 — Schematic diagram of auxin and glutathione synthesis (Moones., 2005). [file Image2.png]
