# Supplementary material for: Glutathione S-transferase CrGST24 in the differentiation of adventitious buds from Camellia reticulata callus
Source: Front Plant Sci. 2025 Aug 15;16:1641401. doi: 10.3389/fpls.2025.1641401 (PMC12395571; doi:10.3389/fpls.2025.1641401)
Supplement: Supplementary Table 1 — The culture medium required for the research. [file Table1.docx]

Supplementary Table 1. The culture medium required for the research

| Medium | Medium ingredients |
| --- | --- |
| YS1 | MS+0.1 mg/L IBA+0.5 mg/L 6-BA+1.0 mg/L KT+2.0 mg/L NAA+5.0 mg/L PVP+3% Sucrose |
| YS2 | MS+0.2 mg/L IBA+1.0 mg/L 6-BA+1.0 mg/L KT+2.0 mg/L NAA+5.0 mg/L PVP+3% Sucrose |
| YS3 | MS+0.1 mg/L IBA+1.0 mg/L 6-BA+1.0 mg/L KT+2.0 mg/L NAA+5.0 mg/L PVP+3% Sucrose |
| YS4 | MS+0.2 mg/L IBA+0.5 mg/L 6-BA+1.0 mg/L KT+2.0 mg/L NAA+5.0 mg/L PVP+3% Sucrose |
| YEB1 | YEB + 100 mg/L Rif + 100 mg/L Kan + 16 g/L Agar |
| YEB2 | YEB + 100 mg/L Rif + 100 mg/L Kan |
| MS1 | 3% MS |
| MS2 | 3 % MS + 2.25 mg/L 6-BA + 0.3 mg/L NAA + 6.5 g/L Agar |
| MS3 | 3 % MS + 2.25 mg/L 6-BA + 0.3 mg/L NAA + 20 mg/L Hyg + 400 mg/L Cef + 6.5 g/L Agar |
| MS4 | 3 % MS + 0.1 mg/L 6-BA +0.1 mg/L NAA + 20 mg/L Hyg + 400 mg/L Cef + 6.5 g/L Agar |
| MS5 | 3 % MS + 20 mg/L Hyg + 200 mg/L Cef + 6.5 g/L Agar |
| AsA1 | 3 % MS + 50 mg/L AsA |
| GSH1 | 3 % MS + 922 mg/L GSH |
| A1G1 | 3 % MS + 50 mg/L AsA + 922 mg/L GSH |
